# An Investigation into the Effects of Organisms upon the Teeth and Alveolar Portions of the Jaw

**Published:** 1882-05

**Authors:** 


### AMERICAN JOURNAL

OF

### DENTAL SCIENCE.

**Vol. XVI. THIRD SERIES—MAY, 1882. No. 1.**

### ARTICLE I.

### *An Investigation into the Effects of Organisms Upon the Teeth and Alveolar Portions of the Jaw.*

The effects of the presence of septic organisms in various parts of the body have been for some years the subject of vigorous discussion and inquiry. Up to the present time certain main facts have, we consider, been placed beyond doubt, and accepted by the general body of scientfic men as incontrovertably established. We shall therefore assume as axioms in our present inquiry, the main principles of the antiseptic theory. The object of our inquiry (undertaken some four yeras ago) has been to determine, as far as possible, to what extent germs are present in the dental tissues when in a state of disease, and to deduce conclusions as to the effects of their presence. The method we have pursued has been threefold;

1. *Microscopical*—The observation of numerous sections of morbid dental tissues, stained in such a manner as to render the germs plainly visible.

2. *Flask Experiments*—The exposure of dental structure in a healthy condition to the action of septic and aseptic fluids.

3. *Clinical Experiments*—The treatment of morbid conditions of the dental tissues with antiseptic agents.

The results of this threefold investigation have uniformly led us to the conviction that germs play an important part in the production and maintenance of morbid conditions of the dental tissues. With regard to the principal calcified dental tissue, dentine, and its destruction by caries, it is necessary to allude, for a moment, to the most commonly received views of the etiology of the latter disease. The old "vital theory" may be passed over, as completely disproved by the successful experiments of M. Magitot and many others, who have produced caries in teeth not in connection with the living body. With regard to the purely chemical theory, we cannot accept it as wholly satisfactory, for the following reasons:—

1. Because the destruction of dentine effected by the action of acids alone, under aseptic conditions, does not resemble caries, either in color or in consistency, it being colorless and gelatinous, the process uniformly attacking all parts of the surface.

2. Because sections of dentine so destroyed show uniform destruction of the matrix, but not enlargement of the channels occupied by the fibrils; whereas, true caries first attacks the soft tissues, *i. e.*, the fibrils, and encroaches from that *point d' appui* upon the surrounding calcified structure, thereby producing the characteristic enlargement of the channels, until two channels break into one, the intervening matrix being wholly destroyed.

3. That although artificial caries has been produced exactly resembling true caries, we have failed to discover any record of an experiment in which this has been the case when septic influences were excluded. Two experiments have, indeed, been recorded in which the teeth were protected from septic agencies, in one by the addition of creasote ("Tomes' Dental Surgery,") in the other by hermetic sealing of the flask, and in neither of these did caries occur. We assume, therefore, that two factors have always been in operation (1,) the action or acids, or (2,) the action of germs. Further, our own flasks shows that malic and butyric acids, with salvia in a meat infusion, have not, under aseptic conditions, produced caries.

It may be asked, if the tooth has been decalcified by acids out of the mouth, and these acids are constantly in action in the mouth, then if they produce caries, why can they not produce simple decalcification? To this it may be replied, that acids alone do not destroy a living tissue; that the stomach is not digested by its own acids until it has been removed from the body.

4. Lastly, we would urge that, when caries occur in the mouth, it is always under circumstances more favorable to the action of germs than to that of acids. There is always, first of all, a minute pit or haven where germs can rest undisturbed and attack the tissue. We cannot, upon the purely acid hypothesis, explain why the same acids that originally caused the decay, gaining access through some minute imperfection of the armor of enamel, do not in the same mouth, or under the same condition, attack the wounded enamel at the edges of the fillings. The germs cannot rest there, they are constantly washed away if the surface is fairly smooth; but the acids literally bathe the part, except during the performance of the act of mastication, when the alkaline parotid and submaxillary salvia neutralize their action.

These considerations led us to seek for signs of the presence of organisms in carious dentine, with results that far exceeded our expectations.

### THE "SEPTIC" THEORY.

This theory—which, for the sake of distinction, may be called "septic"—is rather an amplification of the chemical theory than a contradiction of it. Most probably the work of decalcification is entirely performed by the action of acids, but these acids are, we think, secreted by the germs themselves, and the organic fibril upon which the organisms feed, and in which they multiply, is the scene of the manufacture of their characteristic acids which, in turn, decalcify the matrix, and discolor the whole mass.

From our observation of cementum to which caries has extended, we conclude that the process there is very similar; the bioplasmic contents of the lacunæ and canaliculi afford board and lodging for the organisms, which multiply, and when sufficiently numerous, decalcify the surrounding bone so that the lacuna loses its outlined and extends in all directions.

With regard to the pulp, its inflammation does not materially differ from that of any other cellular connective tissue. The influence of germs upon such tissues, when inflamed, has been already demonstrated by Professor Lister. One peculiarity distinguishes this from kindred tissues, and that is that a tooth being an absolutely shut box, its contents are singularly amenable to antiseptic treatment. Having once rendered them aseptic by means of a penetrating agent, it is easy to keep them absolutely so, by sealing the only opening of the cavity with a filling. With a view of testing this fact, the theoretical accuracy of which we could not doubt, we have repeatedly allowed the gangrenous contents of a pulp cavity in which suppurative inflammation has gone on, to remain in the tooth covered by a filling.

All that is necessary to prevent further disturbance is to soak them in a powerful antiseptic. I have chosen for this purpose a combination of iodoforn and eucalyptus oil, because it is the most powerful and the most permanent in its effects. After a certain period, during which the cavity has been sealed with a temporary filling, if no disturbance has occurred, I have substituted a permanent filling, and almost invariably with the most satisfactory results. The treatment is, I think, eminently conservative surgery and based upon sound surgical principles.

M. Magitot, in a recent most interesting article upon the development of the teeth, lays great stress upon the fact that the odontoblast layer is very essential to the health of the tooth; and if we can, by this method of dealing with the matter, avoid the otherwise necessary alternative of removing it together with all dead matter, we certainly give the tooth and extra chance. The more of the natural vital contents of the pulp cavity we can preserve *in situ* the better for the the tooth.

Further, we would propose for your consideration a theory as to what is the probable course of behavior of a tooth pulp under these circumstances; at present only a theory, but one which we hope to establish shortly by occular demonstration, and lay before you in a mature form. Suppose a tooth, with a large cavity communicating with the pulp-chamber; suppose that gangrene and suppuration have extended down two-thirds of the pulp-chamber, and that the extreme periphery of the pulp (membrana eboris,) and the contents of the apex of the root alone remain alive; suppose the cavity cleared, a small portion of the surface of the slough removed, and the rest carefully soaked with eucalyptus oil until all the existing organisms are destroyed and the cavity then filled with a permanent and effectual stopping, what becomes of the slough? In every other part of the body a slough that has been rendered and kept aseptic is gradually absorbed by the neighboring vessels, while new cicatricial connective tissue is laid down in its place, this has been repeatedly demonstrated in sloughs of the limbs treated antiseptically. We therefore suggest that the same thing that happens in other parts of the body, under similar circumstances, happens in the pulp-chamber; the slough is removed gradually by absorption, while its place is supplied by healthy cicatricial tissue; like all scar tissues, it is of a lower order than that which it replaces, but it is a living tissue. After a lapse of time, then, we imagine that if the tooth in question were extracted and split open, its contents would be found to be a healthy connective tissue, and not a slough at all.

### EXPERIMENTS ON THE LIVING SUBJECT.

Taking, then, for granted the facts established by Lister and others, relative to the effects of the presence of organisms in other tissues, we were convinced that the course of an alveolar abscess was also profoundly modified by their presence. An aseptic or a septic alveolar abscess seemed to us to differ very much in the same way as a simple and a compound fracture.

To render such abscesses aseptic, we have injected them with eucalyptus oil and iodoform, and dressed them with lint soaked in these substances.

By means of constant and careful dressings and injections, we have been for the last two years very successful in inducing extensive, and often old standing alveolar abscess to run a rapid aseptic course of recovery. Wherever we have failed we have been able to trace our failure to the presence of a sequestrum, which has afforded an inaccessible refuge for organisms, and which, until nature has rejected it, or surgical skill removed it, has kept up the mischief despite all applications. Mr. David Hepburn, in a recent able paper read before the Odontological Society, urged this fact very strongly and clearly, and we quite agree with him that, while dead boue is present, antiseptics may mitigate, but are powerless to annihilate the disease.

MICROSCOPICAL.

The sections from which our observations have been made have been cut from fresh teeth, very shortly after extraction, and without the use of any decalcifying or softening re-agent.

We have subsequently stained them with an aniline dye (methyl violee,) following as closely as possible that recommended by Koch—a process which renders micrococci fairly distinct under a $\frac{1}{8}$ lens, with proper illumination. A few typical slides we have been enabled to show you, owning to the kindness of our friend, Mr. Nelson. I need scarcely add that as the powers are very high oil immersions (1–60, 1–32, 1–25,) we shall ask you to refrain from touching the adjustments. We have also prepared some diagrams illustrating upon a larger scale the appearance of the objects. In dentine, which has occupied most of our attention, we have invariabiy discovered the channels containing the dentinal fibrils more or less infiltrated with germs—for the most part micrococci, oval and rod-shaped bacteria. These germs we find penetrating, at first in Indian file, then more thickly, along the course of the fibrils. As they accumulate and choke up the channels they encroach upon the matrix, diminishing the distance between the fibrils until the matrix entirely disappears, the neighboring channels join, and the whole tissues becomes one conglomerated mass of organisms. Beyond the sphere of visible decay, sections cut from apparently healthy tissue show here and there a narrow line of micrococci or bacteria, like an advance guard, and such insolated tubes or germs probably penetrate far into tissue which the naked eye would pronounce sound.

In decay which has appeared in blocks of hippopotamus ivory worn on a plate, we have observed very similar appearances, also in some caries which we have produced ourselves in a flask, by exposing a sound tooth to septic agencies.

In cementum hitherto we have experienced some difficuty in obtaining sections of tissue into which caries has penetrated; but where we have succeeded we have found the lacunæ filled with germs. In some lacunæ the protoplasm of the osteoblast cell is slightly stained, the nucleus very deeply, and a few germs are seen scattered about in little groups. In others the protoplasmic contents of the space appear to have been totally destroyed, the outline lost, and the whole lacuna crowded with germs.

SUMMARY.

The preceding observations, together with the experiments with flasks and upon the living subject above referred to, have less to us to adopt certain views which may be regarded as an extension of the views previously held upon the matter.

1. We consider that caries is absolutely dependent upon the presence and prolification of organisms. That those organisms attack first the organic material, and feeding upon it, create an acid which removes the lime salt, and that all the differences between caries and simple decalcification by acids is due to the presence and operation of germs. This view we propose to call the "septic theory."

2. That suppuration of the pulp and its sequelæ, such as alveolar abscess, depend also upon the successful working of organisms.

3. We feel justified in concluding that the successful exclusion of germs would prevent the disease, and that their exclusion is quite easy and practicable by the use of powerful and penetrating antiseptic agents, such as eucalyptus oil.

We have found that the space of time allotted to a paper rendered it quite impossible to enter into all the details of treatment and experiment we could wished. These experiments have extended over three years, and cannot be condensed into an half-hour paper. All therefore, that we have been able to do has been to lay before you the main facts and a few typical results, and in doing so we have been very kindly assisted by Mr. Nelson, whose microscopical arrangements have enabled us to show you our results in a more perfect manner than would have been otherwise possible.—*Journal of the British Dental Association.*

